# The influence of vertebrate scavengers on leakage of nutrients from carcasses

**DOI:** 10.1007/s00442-024-05608-w

**Published:** 2024-08-17

**Authors:** Elke Wenting, Patrick A. Jansen, Simon Burggraeve, Devon F. Delsman, Henk Siepel, Frank van Langevelde

**Affiliations:** 1 Department of Environmental Sciences, Wageningen University and Research, Box 47, 6700 Wageningen, The Netherlands; 2 Department of Ecology, Radboud Institute for Biological and Environmental Sciences, Radboud University, Box 9010, 6500 Nijmegen, The Netherlands; 3 Smithsonian Tropical Research Institute, Ancon, Balboa, Panama

**Keywords:** Carrion, Decay, Nutrient cycle, Scavengers, Decomposition

## Abstract

The decomposition of carcasses by scavengers and microbial decomposers is an important component of the biochemical cycle that can strongly alter the chemical composition of soils locally. Different scavenger guilds are assumed to have a different influence on the chemical elements that leak into the soil, although this assumption has not been empirically tested. Here, we experimentally determine how different guilds of vertebrate scavengers influence local nutrient dynamics. We performed a field experiment in which we systematically excluded different subsets of vertebrate scavengers from decomposing carcasses of fallow deer (*Dama dama*), and compared elemental concentrations in the soil beneath and in the vegetation next to the carcasses over time throughout the decomposition process. We used four exclusion treatments: excluding (1) no scavengers, thus allowing them all; (2) wild boar (*Sus scrofa*); (3) all mammals; and (4) all mammals and birds. We found that fluxes of several elements into the soil showed distinct peaks when all vertebrates were excluded. Especially, trace elements (Cu and Zn) seemed to be influenced by carcass decomposition. However, we found no differences in fluxes between partial exclusion treatments. Thus, vertebrate scavengers indeed reduce leakage of elements from carcasses into the soil, hence influencing local biochemical cycles, but did so independent of which vertebrate scavenger guild had access. Our results suggest that carcass-derived elements are dispersed over larger areas rather than locally leak into the soil when vertebrate scavengers dominate the decomposition process.

**Supplementary Information:**

The online version contains supplementary material available at 10.1007/s00442-024-05608-w.

## Introduction

The decomposition of dead animal bodies—carcasses—can severely alter the local soil chemical composition and biochemical cycles (e.g.,Carter et al. 2007; Barton et al. 2019; Quaggiotto et al. 2019; Newsome et al. 2021). Despite its relatively small contribution of approximately 1% to the total detritus pool, carcass decomposition is pivotal in imparting limiting nutrients within ecosystems (e.g.,Parmenter & MacMahon 2009; Barton et al. 2016; Wenger et al. 2019). We use the term ‘nutrients’ when the chemical elements serve as food particles, the term ‘minerals’ when elements are still bound in soil chemical compounds and the term ‘(chemical) elements’ in a general more neutral way. Existing studies have predominantly focused on a restricted number of chemical elements in relation to carcass decomposition (e.g.,Barton et al. 2013; Monk and Schmitz 2022). Consequently, our understanding of the role of carcass decomposition in nutrient cycles, based on a wide variety of essential nutrients that are crucial for all lifeforms (e.g.,Robinson et al. 2009; Kaspari 2021), is limited.

The extent to which carcass decomposition alters the local biochemical cycles highly depends on the interplay between scavengers—both vertebrates and invertebrates—and microbial decomposers (Carter et al. 2007; Stiegler et al. 2020). The outcome of the interplay itself is subject to a range of abiotic factors including ambient temperature, and biotic factors including the composition of the scavenger guild (e.g., Meyer et al. 2013; Farwig et al. 2014; Feddern et al. 2019; Olea et al. 2019; Wenting et al. 2022). Carcass consumption by vertebrate scavengers, in particular, would play a key role in dispersing carcass-derived nutrients over larger areas, resulting in a reduced impact on local nutrient dynamics (e.g.,DeVault et al. 2003; Melis et al. 2007; Benbow et al. 2019; Subalusky & Post 2019). Contrary, carcass decomposition dominated by microbial decomposers and invertebrate scavengers would have enlarged effects on local nutrient dynamics (Janzen 1977).

Different scavenger species, particularly vertebrates, are assumed to differently influence elemental fluxes because they differently influence the carcass decomposition speed (e.g.,Wenting et al. 2022; Bartel et al. 2024). This is mainly because birds and mammals exploit carcasses in different ways (e.g., Moleón et al. 2014; Patterson et al. 2022). In the absence of obligate scavengers such as vultures, wild boar (*Sus scrofa*), in particular, can greatly speed up the carcass decomposition process (Wenting et al. 2022). Vertebrates can, in general, consume larger amounts in short time periods compared to invertebrates and can so disperse carcass-derived elements over larger areas (e.g.,DeVault et al. 2003; Sebastián-González et al. 2016; Morales-Reyes et al. 2018). Carcass decomposition dominated by invertebrates might proceed slower (e.g. Zanetti et al. 2015), although delayed insect access can decrease decomposition rates as well (Pechal et al. 2014). However, the impact that different scavenger guilds can have on local nutrient dynamics remains unknown.

It has been demonstrated that carcass decomposition can lead to increased elemental leakage into the soil (e.g. Barton et al. 2019; Taylor et al. 2023; Wenting et al. 2023a). However, so far, studies have focused on a limited number of chemical elements. For example, carcass decomposition can alter the soil chemical composition by increasing concentrations of C and N, coupled with changes in pH (Macdonald et al. 2014; Keenan et al. 2018; Quaggiotto et al. 2019). Concentrations of P can slightly increase as soft tissues decompose (e.g. Melis et al. 2007; Barton et al. 2016; Wenger et al. 2019; Heo et al. 2021). Other chemical elements, e.g. Ca, can be both released in early decomposition stages as well as cause a long-lasting substantial increase due to slow decomposition of skeletal remains (e.g., Taylor et al. 2023).

Carcass decomposition is believed to cause local nutrient pulses (Yang et al. 2008). However, comprehensive overviews of elemental changes during the decomposition process are scarce. Some studies measured electrical conductivity (EC) below decomposing carcasses, as a proxy for multiple elemental changes, e.g., Na and K (e.g.,Keenan et al. 2018; Quaggiotto et al. 2019). Yet, EC does not provide any insights into the changes of individual elemental concentrations. Others considered Ca, K, Na, Mg, and S below carcasses. They found pulses in K, Na, and S concentrations that gradually dissipated over time, whereas Ca and Mg showed a continuous release over longer periods (e.g., Parmenter and MacMahon 2009). Wenting et al. (2023a) provided the most comprehensive overview to date, measuring 22 chemical elements, but did not measure changes over time. Taylor et al. (2023) measured 14 elements over time, but did not study the role of vertebrate scavengers. Thus, the magnitude of alleged pulses of elements leakage from decomposing carcasses over time, influenced by vertebrate scavengers, remains unknown.

Carcass-derived elements can leak into the soil in concentrations that enhance plant growth (Barton et al. 2016; Wenting et al. 2023a), which can in turn influence foraging behaviour and movement patterns of animals, hence overall ecosystem dynamics (e.g.,Doughty et al. 2016; Pringle et al. 2023). Nutrient pulses can result in the formation of islands of fertility (Zaady et al. 1996; Carter et al. 2007). When plants grow on the edges of decomposing carcasses and take up the available nutrients, plant quantity or plant quality is expected to increase. Investment in quantity results in increased plant growth, i.e., biomass (e.g.,Towne 2000; Danell et al. 2002). Investment in quality results in increased elemental concentrations in plant tissues (e.g., Pilon-Smits et al. 2009). Melis et al. (2007) described that the effects on plant nutrient concentrations are not easily detectable in temperate ecosystems. To date, however, there are no studies examining the elemental concentrations in plants close to carcasses, including a wide range of chemical elements.

This study aimed to experimentally determine how different guilds of vertebrate scavengers influence local nutrient dynamics in a protected area without obligate vertebrate scavengers. We hypothesized that vertebrate scavengers, particularly wild boar, can take up the majority of the carcass-stored elements in their bodies, and that local leakage of elements will thus be greater as more vertebrate scavengers are excluded. We investigated this with a field experiment in which we systematically excluded different subsets of vertebrate scavengers from decomposing carcasses, and compared elemental concentrations in the soil beneath and in the vegetation next to the carcasses over time throughout the decomposition process.

## Methods

### Study area

The experiment was performed at Veluwezoom National Park (henceforth ‘Veluwezoom’), the Netherlands (52°02′N, 6°01′E). Veluwezoom, a former agro-silvopastoral landscape (Kuiters 2005), is a protected area of 5000 ha situated partly on glacier deposits and partly on cover sands, characterised by sandy and loess soils. This characterizes the natural mineral availability as limited to very scarce, especially on the dominant sandy soils. The area contains a mosaic of dry grass-heathlands, pastures, abandoned crop fields and woodland. Natural processes with minimal human interference are of major importance in the management strategy (Kuiters 2005), in which a key role is played by free-ranging Scottish highland cattle (*Bos taurus*), Icelandic horses (*Equus ferus caballus*), fallow deer (*Dama dama*), red deer (*Cervus elaphus*) and wild boar (Bruinderink & Lammertsma 2001). Veluwezoom inhabits several facultative vertebrate scavengers, including wild boar, European pine marten (*Martes martes*), European badger (*Melis melis*), common raven (*Corvus corax*), and common buzzard (*Buteo buteo*) (Wenting et al. 2022).

### Experimental design

**Fig. 1:**
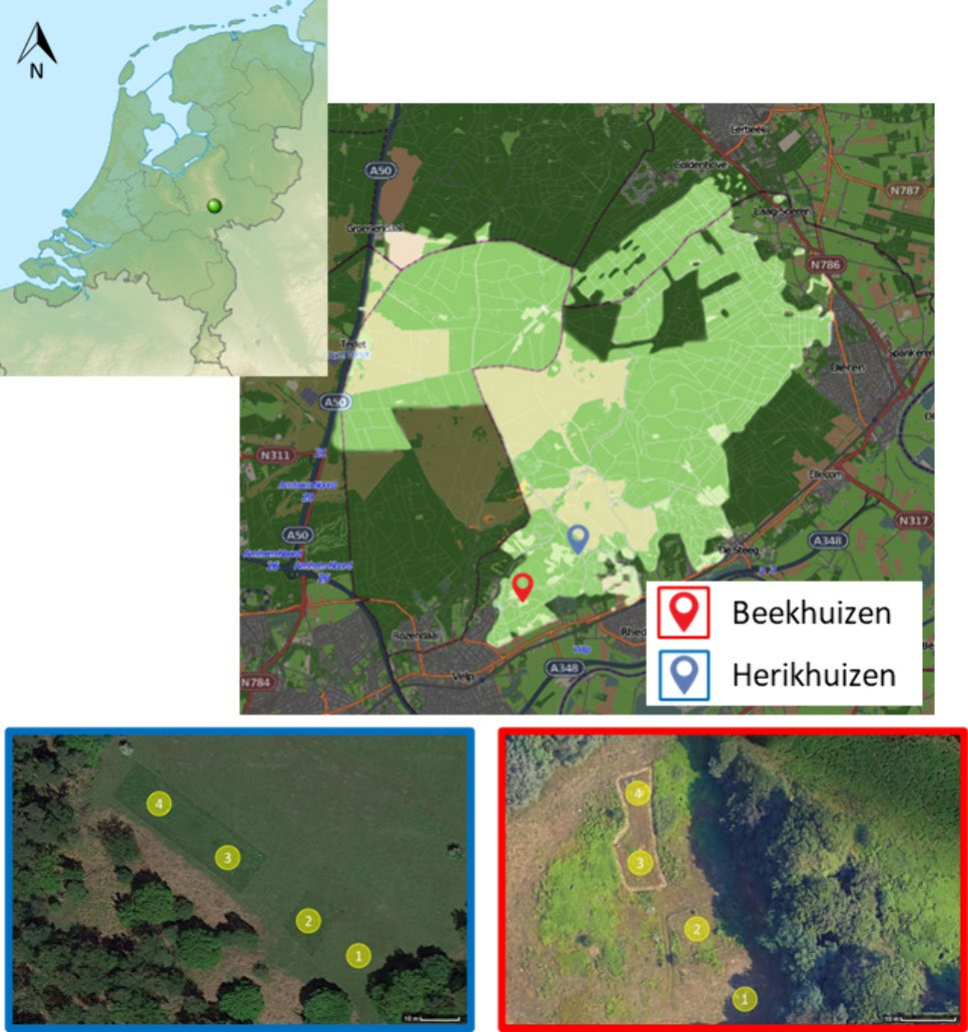
Map showing the location of Veluwezoom National Park, the Netherlands. The locations of the study areas (Beekhuizen and Herikhuizen) are also indicated, including how the locations where the exclusion treatments were executed: (1) allowing all scavengers; (2) excluding wild boar (*Sus scrofa*); (3) excluding all mammalian scavengers; and (4) excluding all vertebrate scavengers. See Fig. 2 and Appendix 1 for photos of the experimental design

We performed the experiment at two locations in the southern part of Veluwezoom (Fig. 1): Beekhuizen (52°00′35.1′′N, 5°59′34.8′′E) and Herikhuizen (52°01′03.7′′N, 6°00′25.3′′E), in which we followed the decomposition of fresh carcasses of fallow deer. The carcasses were obtained from regular culling, i.e., no animals were killed for the purpose of our study. The carcasses originated from the same areas as described by Wenting et al. (2023b; c). Fallow deer is a terrestrial herbivorous ungulate with an adult body weight of 40–80 kg and a non-nomadic lifestyle (Focardi et al. 2006). The carcasses in our experiment were adult females with a body mass ranging from 40 to 50 kg.

**Fig. 2:**
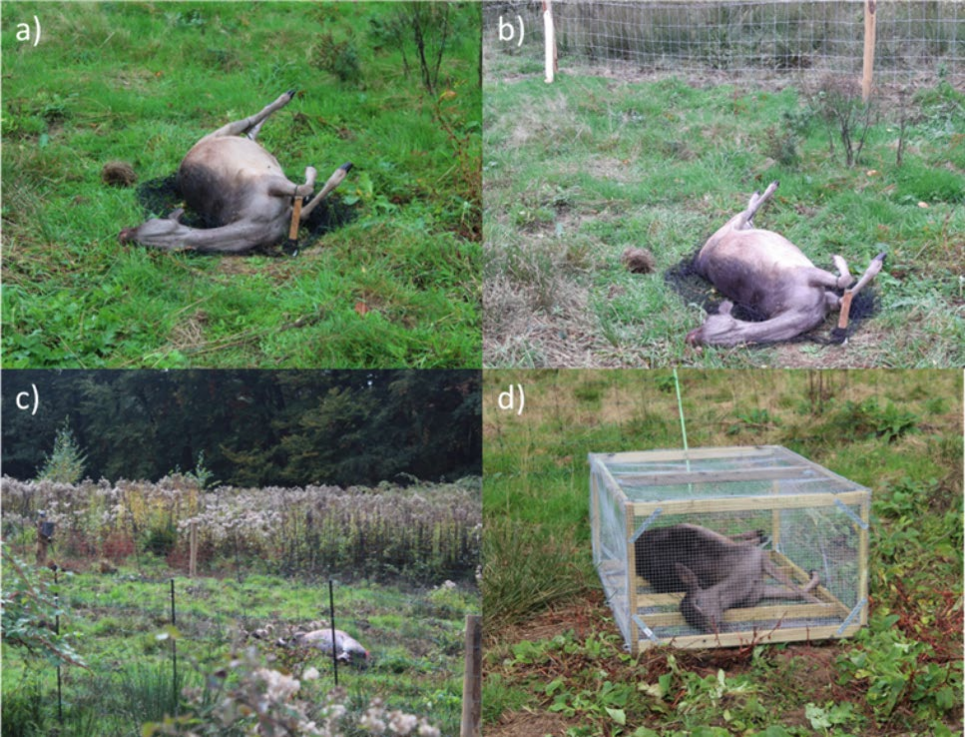
Photos of a fallow deer (*Dama dama*) carcass in each of the scavenger exclusion treatments: **a** allowing all scavengers; **b** excluding wild boar with a firm fence; **c** excluding all mammalian scavengers with an electricity fence; and **d** excluding all vertebrate scavengers using a cage

Each experimental trial consisted of three or four treatments (Table 1), i.e., we needed four carcasses per trial. These treatments differed in the subset of scavenger guilds that got access to the carcasses: (1) no scavengers excluded; (2) wild boar excluded; (3) all mammalian scavengers excluded; and (4) all vertebrates excluded, i.e. mammals as well as birds. The carcasses in the first treatment were unprotected, i.e., allowing all scavengers (Fig. 2a). For the second treatment, we used a firm fence to exclude wild boar from the carcasses, with a height of 120 cm and mesh sizes ranging from 8 cm at the bottom to 13 cm at the top (Fig. 2b). The carcasses in the third and fourth treatment were placed in exclosures with electric fences, with a voltage of at least 5.5 V (Fig. 2c). Cages excluded avian scavengers in the fourth treatment (Fig. 2d). It was physically impossible for other scavengers than intended to reach the carcasses in each treatment. These treatments were based on the scavenger guilds described by Wenting et al. (2022), in which Veluwezoom was one of the study areas. In a pilot study we also tried to exclude all invertebrate scavengers as a fifth treatment, but this appeared impossible for practical reasons.

**Table 1:** Overview of the experimental trials

Trial	Location	Start date	Sampling weeks	No. of carcasses	Notes
Trial 1	Beekhuizen	15th Oct ‘19	0–4–8–12–16–20	4	Sampling not continued due to pandemic
Trial 2	Herikhuizen	4th Feb ‘20	0	4	Electricity fence destroyed by wild boar, excluded from analyses
Trial 3	Herikhuizen	8th Sept ‘20	0–3–8–12–17–21–24–27–32–36–40	4	
Trial 4	Beekhuizen	3rd Nov ‘20	0–4–9–13–16–19–24–28–32	4	
Trial 5	Beekhuizen	14th Sept ‘21	0–4–9–13–17–21–25–29–33–37–40	4	
Trial 6	Herikhuizen	26th Oct ‘21	0–3–7–11–15–19–23–27–31–34–38	4	
Trial 7	Beekhuizen	8th March ‘22	0–4–8–12–15–19–23–28–32–36–40	3	Only treatment 2–4 due to management change
Trial 8	Herikhuizen	23th Aug ‘22	0–4–7–12–16–20–24–27–32–36–40	4	
Trial 9	Beekhuizen	13th Sept ‘22	0–4–9–13–17–21–24–29–33–37–40	3	Only treatment 2–4 due to management change

Each trial started at the same day, meaning that we needed four carcasses simultaneously. Carcasses were randomly assigned to one of the treatments. The carcasses were placed about 30 m apart from each other at one of the two locations. All carcasses were placed with their abdomen to the south. For the first three treatments, carcasses were placed on top of a net (to lift them, see data collection section) and tied by the front and rear legs to poles of 50 cm above the ground to prevent dragging. Carcasses in the fourth treatment were placed in a cage made of 1 × 1 cm chicken wire. All carcasses were placed in direct contact with the soil.

Using two locations allowed us to run two experimental trials at the same time. We repeated the experiment nine times in total (Table 1) between 15 October 2019 and 13 September 2022. However, due to the pandemic and failing electricity fences, we could only partially include the first trial and excluded the second trial from the analyses, meaning that we used seven trials fully and one trial partially in the entire experiment. The last carcasses were sampled on 20th of June 2023. In total we used 34 carcasses for the entire study, of which 4 carcasses (“Trial 2”) could not be monitored due to failing exclusion constructions.

### Data collection and measurements

We followed the decomposition process of each trial for about 40 weeks, with sampling intervals of 4 ± 1 weeks. The first sample (*t* = 0) was taken at the day the experimental trial started. We collected three sample types per moment of sampling: (1) the first 5 cm of the soil; (2) roots, i.e., below-ground plant parts; and (3) shoots, i.e., above-ground plant parts. The root and shoot samples were taken as a combined vegetation sample and mainly consisting of grasses. The soil samples were taken directly beneath the carcasses and the vegetation samples were taken at the direct edges of the carcasses. We chose the plants closest to the carcasses, including the ones that were visibly affected by the carcasses, to evaluate the most realistic situation. Thus, we did not aim to only collect visibly healthy or affected plants specifically but collected vegetation samples dependent on the local conditions. To access the soil, we lifted the carcass using a transportable scaffolding (Appendix 1). After sampling, the scaffolding allowed us to put the carcasses back in exactly the same position as before.

For each sample type, a control sample was taken at approximately 1 m distance from the carcass. This distance was based on previous studies, which found no or only very limited lateral spread beyond 30 cm from carcasses (Melis et al. 2007; Keenan et al. 2019; Barton et al. 2020).

All samples were dried for at least 48 h starting on the day of sampling: the soil samples at 40 °C and the vegetation samples at 70 °C. After drying, we separated the roots and shoots from the vegetation samples using sieves and tweezers. The soil samples were homogenized using sieves. All the samples were stored in paper bags until chemical analysis in the laboratories of Radboud University.

We used a microwave digestion method with 5 ml 65% nitric acid (HNO_3_) and 2 ml hydrogen peroxide (H_2_O_2_) to prepare all the samples for measuring the elemental concentrations. We used Inductively Coupled Plasma Optical Emission Spectroscopy (ICP-OES) to measure nine elements: Al, Ca, Fe, K, Mg, Na, P, S, and Si. Another 12 elements were measured using Inductively Coupled Plasma Mass Spectroscopy (ICP-MS): As, Cd, Co, Cr, Cu, Mn, Mo, Ni, Pb, Se, Sr, and Zn.

### Statistical analyses

All statistical analyses were done in R version 4.3.1 (R Core Team 2023) in four steps. First, the control samples were used to test whether the local conditions at our study sites changed over time. We used linear mixed-effects (LMM) models to check whether the corresponding element showed a different trend over time, depending on the scavenger exclusion treatments. We used the elemental concentration as dependent variable, the interaction between sampling week and treatment as independent variable, and the experimental trial as random factor. The step-up procedure (Benjamini and Hochberg 1995) was then used to account for multiple testing.

Second, elements were selected for further analyses. Therefore, we calculated the mean concentrations of elements in the soil control samples that did not show a significant pattern over time. We also estimated the mean elemental concentrations in fallow deer, summing up the concentrations reported by Wenting et al. (2023b). Elements with higher concentrations in the soil than in fallow deer were excluded. Then, we selected elements with a described function for both plants and animals, based on the literature. The remaining elements were classified as trace or macro element based on Robinson et al. (2009) and Kaspari (2021).

Third, we used Mann–Whitney *U* tests to determine whether the initial concentrations of the selected elements differed between the control samples and the samples taken at the carcass site just before placing the carcasses, i.e., the starting situation of the carcass site and its close vicinity where the control samples were taken. This enabled us to determine whether the elemental concentrations beneath the carcasses differed from the control sites or not. When the starting situation was equal, we assumed that changes over time were due to the carcass placement. Thus, this determined how we interpreted the potential patterns over time as described in the next step.

Finally, we used linear mixed-effects models (LMMs) to test whether the concentrations of the selected elements showed different trends over time, depending on the different scavenger exclusion treatments. The analyses were done for each of the three sample types—soil, root and shoot—separately. Standardized concentrations were used in these analyses, with the values of week 0 set to 1. Values lower than 1 thus indicated a decrease in elemental concentration over time, values higher than 1 indicated an increase. We used scatterplots with trendlines to visualise the trends for each element over time. For the soil samples, we also analysed the pH.

We reported only the most relevant test statistics in the text (see Appendices 2–4 for a full overview of the test statistics). In general, besides providing the quantitative statistics, we also describe visible patterns that might provide inspiration for further testing. In addition, we only tested for linear trends, even though non-linear relations might exist. Additional to the statistical analyses, we, therefore, described whether we observed any patterns over time, although these might not be statistically significant.

## Results

### Control samples

The control samples showed no significant pattern over time for any of the sample types, nor for any of the scavenger exclusion treatments in the control samples (Appendix 2). Therefore, we were able to use the control samples of the soil to calculate the mean elemental concentrations in the soil at our study sites. No elements were thus removed from further analyses based on the analyses of the control samples.

### Selection of elements

Comparing the elemental concentration in the soil, based on the control samples, to the elemental concentration in fallow deer, we found that the elemental concentration in the soil was higher than the concentration in fallow deer for seven of the elements: Al, As, Co, Cr, Fe, Pb, and Si (Table 2). For all the other elements, the concentration in fallow deer was higher than the concentration in the soil.

**Table 2:** Selection of the elements for further analyses

Element	Soil	Deer	Comment	Selected
Al	7057 ± 2658	143 ± 62.78	Soil > deer	No
As	4.749 ± 1.481	0.421 ± 0.081	Soil > deer	No
B	4.181 ± 2.496	89.40 ± 75.997	Essential for plants and animals (Pizzorno 2015; Abdelnour et al. 2018)	Yes, trace
Ca	991 ± 729	504,309 ± 19,223	Essential for plants and animals (Kaspari 2021)	Yes, macro
Cd	0.311 ± 0.197	24.58 ± 27.59	Highly toxic heavy metal (e.g., Patra et al. 2006)	No
Co	5.103 ± 3.305	1.242 ± 1.192	Soil > deer	No
Cr	15.76 ± 4.528	10.22 ± 5.612	Soil > deer	No
Cu	14.75 ± 6.765	487 ± 105	Essential for plants and animals (Robinson et al. 2009)	Yes, trace
Fe	7609 ± 2194	5879 ± 1672	Soil > deer	No
K	1545 ± 2889	257,217 ± 23,951	Essential for plants and animals (Kaspari 2021)	Yes, macro
Mg	915 ± 422	25,639 ± 2143	Essential for plants and animals (Kaspari 2021)	Yes, macro
Mn	424 ± 310	5087 ± 3098	Essential for plants and animals (Robinson et al. 2009)	Yes, trace
Mo	1.267 ± 1.649	22.71 ± 5.332	Essential for plants and animals (Robinson et al. 2009)	Yes, trace
Na	141 ± 109	153,425 ± 11,627	Essential for animals, no function for plants (Kaspari 2021)	No
Ni	6.292 ± 1.784	8.543 ± 5.052	Essential for plants and animals (Robinson et al. 2009)	Yes, trace
P	1429 ± 643	426,028 ± 20,256	Essential for plants and animals (Kaspari 2021)	Yes, macro
Pb	43.60 ± 19.51	11.55 ± 2.811	Fallow deer < Soil	No
S	628 ± 437	193,660 ± 7012	Essential for plants and animals (Kaspari 2021)	Yes, macro
Se	2.873 ± 1.721	9.062 ± 2.656	Essential for animals, no function for plants (Robinson et al. 2009)	No
Si	3359 ± 1988	1435 ± 273	Soil > deer	No
Sr	6.865 ± 2.671	188 ± 28.55	Not essential for plants or animals (Robinson et al. 2009)	No
Zn	58.47 ± 70.77	1444 ± 116	Essential for plants and animals (Robinson et al. 2009)	Yes, trace

The elemental concentrations in the soil are based on the control soil samples. The elemental concentrations in fallow deer (“deer” in table) are based on the concentrations reported in Wenting et al. (2023b). Mean concentrations (ppb) with standard error are reported. The division in trace and macro elements is based on Robinson et al. (2009) and Kaspari (2021)

According to Robinson et al. (2009), five of the remaining elements could be classified as essential trace elements for both plants and animals: Cu, Mn, Mo, Ni, and Zn. They classified B as essential for plants only (Robinson et al. 2009), but recent studies proved B to be essential for animals as well (Pizzorno 2015; Abdelnour et al. 2018). Another five of the remaining elements could be classified as essential macro elements for both plants and animals: Ca, K, Mg, P, and S (Kaspari 2021).

Summarizing, we selected eleven elements on which we focused in the further analyses (Table 2). These included six trace elements (B, Cu, Mn, Mo, Ni, and Zn) and five macro elements (Ca, K, Mg, P, and S).

### Initial concentrations

None of the initial elemental concentrations (at week 0) significantly differed from the elemental concentration in the control sample, for none of the sample types (Appendix 3). For that reason, we assumed that all (potential) trends over time that we found in the next sections could be attributed to the different scavenger exclusion treatments. In the following sections, we used indices as standardized concentrations per experimental trial (week 0 as value 1) because we sampled the carcasses repeatedly over time.

### Soil

**Fig. 3:**
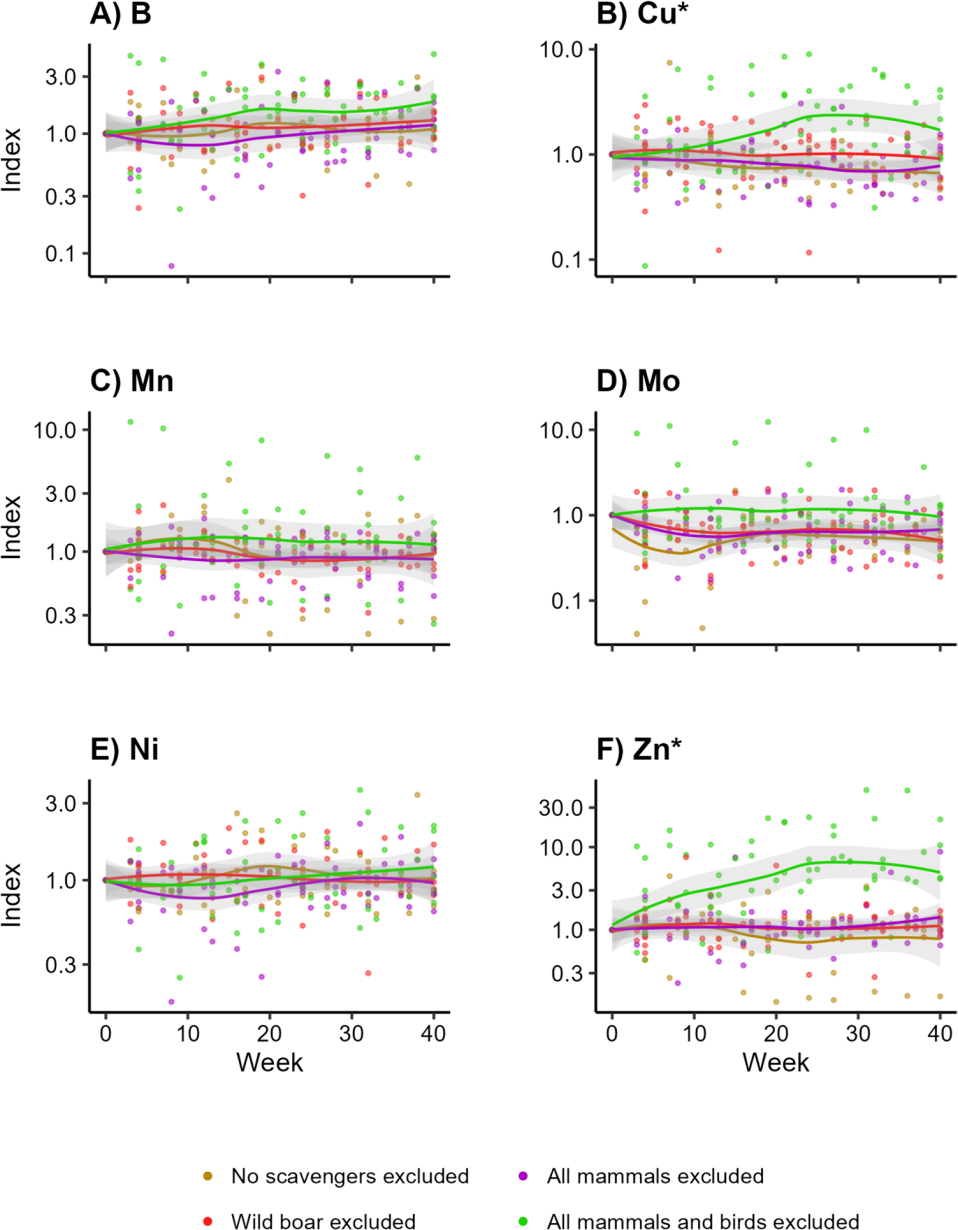
Concentrations of trace elements over time into the soil beneath decomposing carcasses, when different subsets of scavengers are excluded. Visualized as the standardized concentrations per trial, with week 0 as value 1. *Indicates which elements show a significant pattern over time

The soil pH did not change over time (Appendix 4) for any of the treatments. For all trace elements, the treatment that excluded all vertebrate scavengers showed the highest concentrations (Fig. 3a–f). For Cu, soil concentrations peaked at approximately 25 weeks (Fig. 3b; LMM, *df* = 3, *F* = 5.917, *p* = 0.004). The same applied to Zn (Fig. 3f; LMM, *df* = 3, *F* = 7.795, *p* < 0.001). For both elements, the treatment excluding all vertebrates differed from the other treatments (Table 3). This treatment also showed the highest values for Mn (Fig. 3c; LMM, *df* = 3, *F* = 0.032, *p* > 0.99), Mo (Fig. 3d; LMM, *df* = 3, *F* = 0.051, *p* > 0.99), and—to a lesser extent—B (Fig. 3a; LMM, *df* = 3, *F* = 0.587, *p* > 0.99). We did not observe any pattern for Ni (Fig. 3e; LMM, *df* = 3, *F* = 1.357, *p* > 0.94). Moreover, we did not find any pattern for the other treatments that excluded different subsets of vertebrate scavengers (Appendix 4).

**Table 3:** Test statistics belonging to the Tukey post hoc tests of the statistically significant elements in the soil (a) and root (b) samples, indicating which treatments cause the significant trends over time

		Estimate	SE	*df*	*t*.ratio	*p* value
*(a) Soil*						
Element: Cu						
Treatment 1	Treatment 2	−0.211	0.207	256	−1.020	0.738
Treatment 1	Treatment 3	0.054	0.204	257	0.264	0.994
Treatment 1	Treatment 4	−1.251	0.203	256	−6.171	< 0.001
Treatment 2	Treatment 3	0.265	0.188	253	1.409	0.495
Treatment 2	Treatment 4	−1.040	0.188	252	−5.532	< 0.001
Treatment 3	Treatment 4	−1.305	0.184	252	−7.104	< 0.001
Element: Zn						
Treatment 1	Treatment 2	−0.270	0.833	264	−0.324	0.988
Treatment 1	Treatment 3	−0.381	0.829	265	−0.459	0.968
Treatment 1	Treatment 4	−6.025	0.829	264	−7.269	< 0.001
Treatment 2	Treatment 3	−0.111	0.753	259	−0.147	0.999
Treatment 2	Treatment 4	−5.755	0.757	258	−7.599	< 0.001
Treatment 3	Treatment 4	−5.644	0.747	258	−7.555	< 0.001
*(b) Root*						
Element: Cu						
Treatment 1	Treatment 2	0.369	0.627	240	0.588	0.936
Treatment 1	Treatment 3	0.330	0.630	241	0.523	0.953
Treatment 1	Treatment 4	−2.751	0.652	240	−4.220	< 0.001
Treatment 2	Treatment 3	−0.039	0.573	239	−0.068	0.999
Treatment 2	Treatment 4	−3.119	0.598	239	−5.212	< 0.001
Treatment 3	Treatment 4	−3.080	0.594	239	−5.185	< 0.001

Treatments: (1) allowing all scavengers; (2) excluding wild boar (*Sus scrofa*); (3) excluding all mammalian scavengers; and (4) excluding all vertebrate scavengers

**Fig. 4:**
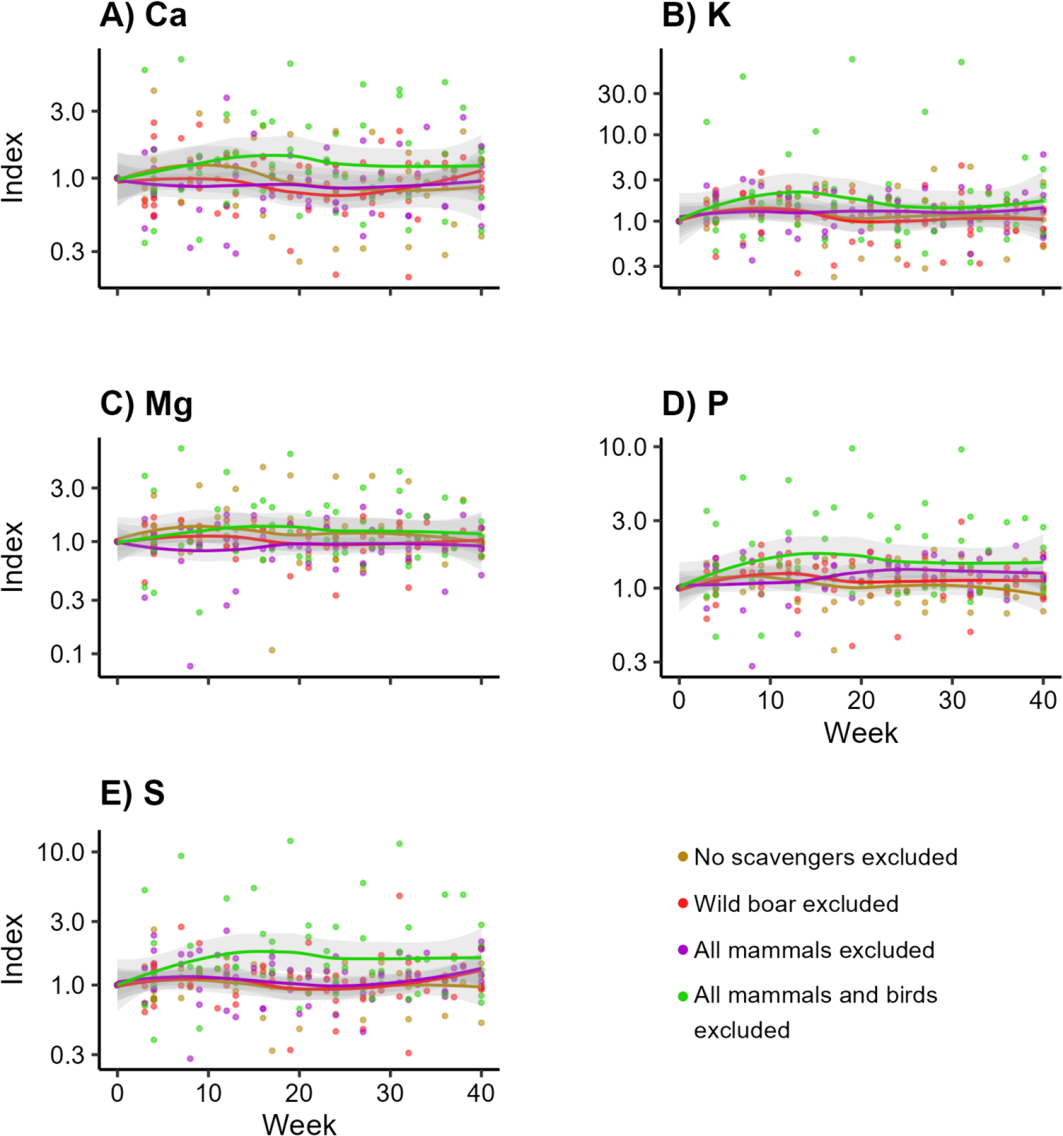
Concentrations of macro elements over time into the soil beneath decomposing carcasses, when different subsets of scavengers are excluded. Visualized as the standardized concentrations per trial, with week 0 as value 1

Similarly, for all macro elements, the treatment excluding all vertebrate scavengers showed the highest values over time (Fig. 4a–e). This was most clear for K (Fig. 4b; LMM, *df* = 3, *F* = 0.040, *p* > 0.99), P (Fig. 4d; LMM, *df* = 3, *F* = 0.550, *p* > 0.99), and S (Fig. 4e; LMM, *df* = 3, *F* = 0.537, *p* > 0.99). The highest values of Mg were attributed to the treatments excluding no scavengers and excluding all vertebrate scavengers (Fig. 4c; LMM, *df* = 3, *F* = 0.029, *p* > 0.99). The trendline of the treatment excluding all vertebrates tended to be higher for Ca, although we did not observe a clear pattern (Fig. 4a; LMM, *df* = 3, *F* = 0.420, *p* > 0.99). However, none turned out significant (Appendix 4).

### Roots

**Fig. 5:**
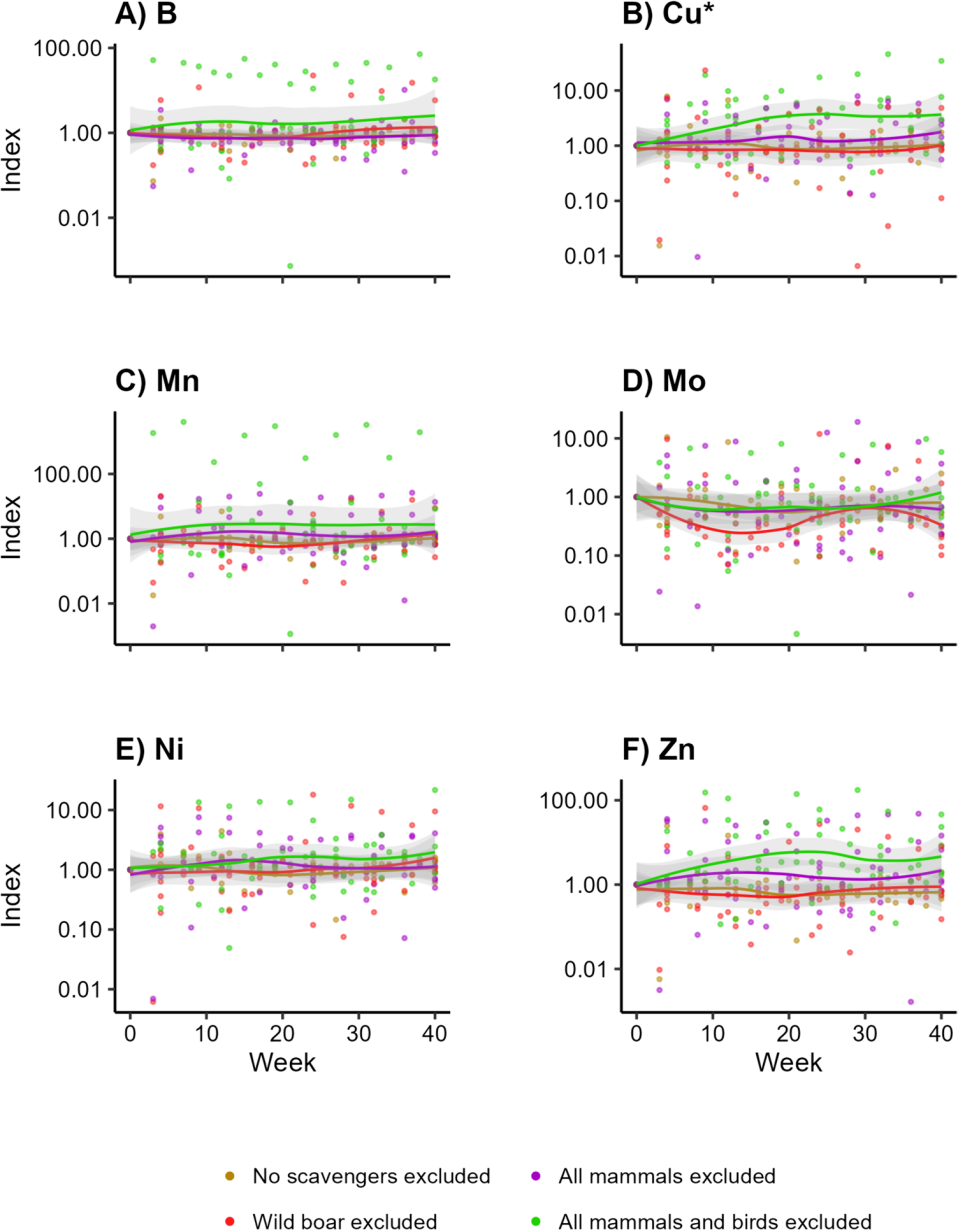
Concentrations of trace elements over time in below-ground vegetation parts (roots) on the edge of decomposing carcasses, when different subsets of scavengers are excluded. Visualized as the standardized concentrations per trial, with week 0 as value 1. *Indicates which elements show a significant pattern over time

For all the root samples, the trendline of the treatment excluding all vertebrate scavengers was generally higher for B (Fig. 5a; LMM, *df* = 3, *F* = 0.303, *p* > 0.90), Cu (Fig. 5b; LMM, *df* = 3, *F* = 7.234, *p* < 0.001), Mn (Fig. 5c; LMM, *df* = 3, *F* = 0.007, *p* > 0.99), and Zn (Fig. 5f; LMM, *df* = 3, *F* = 0.347, *p* > 0.90). Especially for B and Mo, the highest values were attributed to this treatment. We observed most fluctuations for the treatment excluding wild boar for Mo (Fig. 5d; LMM, *df* = 3, *F* = 0.690, *p* > 0.76). There was no clear pattern for Ni (Fig. 5e; LMM, *df* = 3, *F* = 1.310, *p* > 0.57). Only Cu turned out significant, with the treatment excluding all vertebrate scavengers being different from the other treatments (Table 3).

**Fig. 6:**
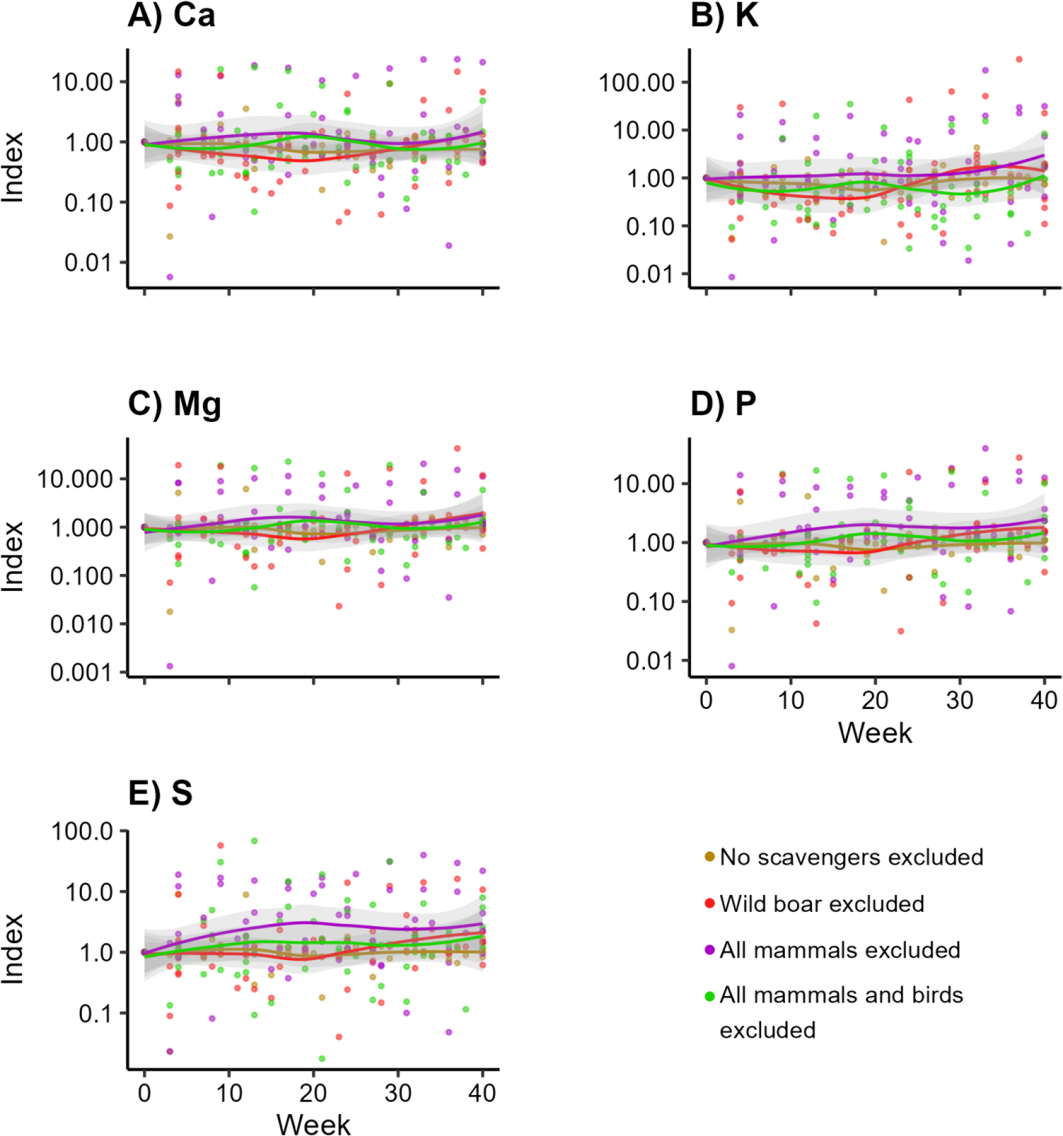
Concentrations of macro elements over time in below-ground vegetation parts (roots) on the edge of decomposing carcasses, when different subsets of scavengers are excluded. Visualized as the standardized concentrations per trial, with week 0 as value 1

Overall, the treatment excluding all mammals tended to be slightly higher compared to the other treatments (Fig. 6a–e), especially for S (Fig. 6e; LMM, *df* = 3, *F* = 1.197, *p* > 0.57) and, to a lesser extent, P (Fig. 6d; LMM, *df* = 3, *F* = 2.015, *p* > 0.57). We did not observe any potential pattern for Ca (Fig. 6a; LMM, *df* = 3, *F* = 1.191, *p* > 0.57), K (Fig. 6b; LMM, *df* = 3, *F* = 1.609, *p* > 0.57), or Mg (Fig. 6c; LMM, *df* = 3, *F* = 0.948, *p* > 0.65). Accordingly, none of the macro elements showed a significant pattern over time in the root samples (Appendix 4).

### Shoots

**Fig. 7:**
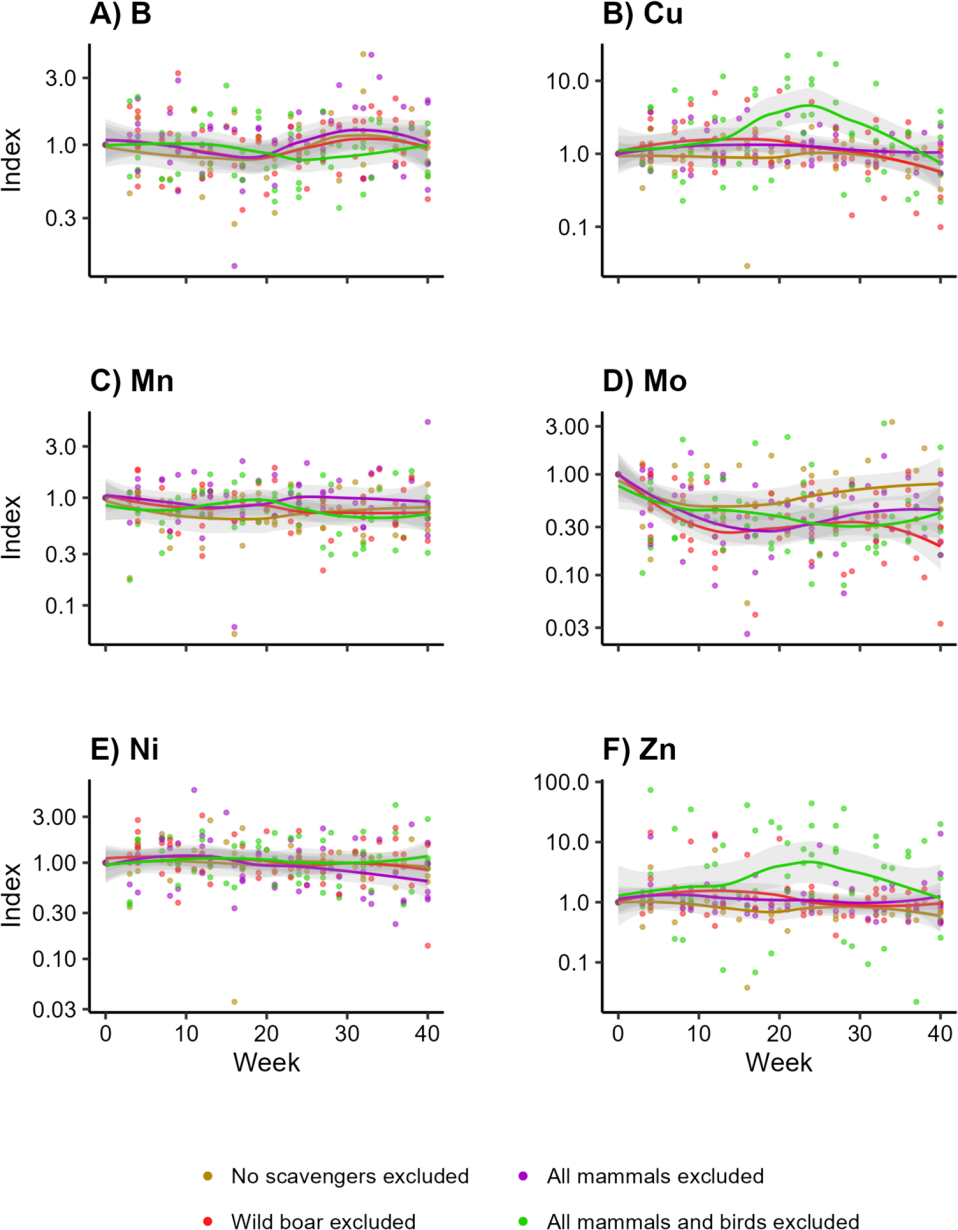
Concentrations of trace elements over time in above-ground vegetation parts (shoots) on the edge of decomposing carcasses, when different subsets of scavengers are excluded. Visualized as the standardized concentrations per trial, with week 0 as value 1

We observed a pulse in Cu (Fig. 7b; LMM, *df* = 3, *F* = 0.709, *p* > 0.60) and Zn (Fig. 7f; LMM, *df* = 3, *F* = 0.050, *p* > 0.98) in the shoot samples for the treatment excluding all vertebrate scavengers, reaching a peak at approximately 22 weeks. Only for Mo, all the trendlines seemed to slightly decrease, with the treatment excluding no scavengers approaching its start level again at the end of the sampling period (Fig. 7d; LMM, *df* = 3, *F* = 3.896, *p* = 0.053). We did not observe any potential pattern for B (Fig. 7a; LMM, *df* = 3, *F* = 1.631, *p* = 0.287), Mn (Fig. 7c; LMM, *df* = 3, *F* = 1.051, *p* = 0.453), or Ni (Fig. 7e; LMM, *df* = 3, *F* = 1.207, *p* = 0.423).

**Fig. 8:**
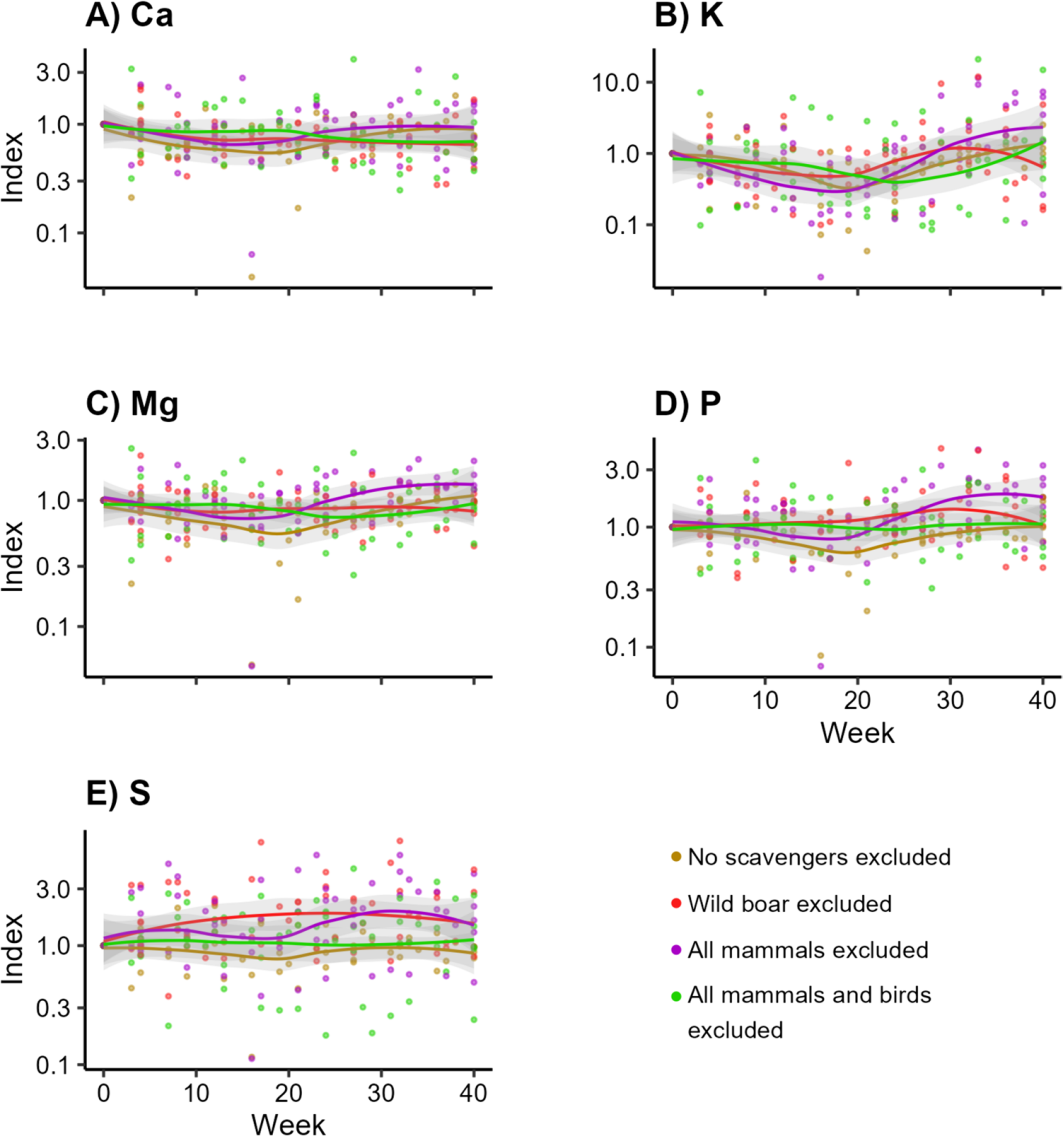
Concentrations of macro elements over time in above-ground vegetation parts (shoots) on the edge of decomposing carcasses, when different subsets of scavengers are excluded. Visualized as the standardized concentrations per trial, with week 0 as value 1

The trendlines of the macro elements tended to end higher at the end of the sampling period for the treatment excluding all mammals for K (Fig. 8b; LMM, *df* = 3, *F* = 2.418, *p* = 0.184), Mg (Fig. 8c; LMM, *df* = 3, *F* = 3.570, *p* = 0.054), and P (Fig. 8d; LMM, *df* = 3, *F* = 4.062, *p* = 0.053). We did not observe any potential pattern for Ca (Fig. 8a; LMM, *df* = 3, *F* = 1.717, *p* > 0.28) or S (Fig. 8e; LMM, *df* = 3, *F* = 1.714, *p* > 0.28). Although for the latter, the treatment excluding wild boar and the treatment excluding all mammals tended to end slightly higher compared to the other two treatments.

## Discussion

Vertebrate scavengers are believed to accelerate elemental cycles by preventing leakage of elements from carcasses into the soil, but this idea had never been empirically tested. We determined how exclusion of different scavenger guilds influenced local nutrient dynamics during carcass decomposition. We found that experimental exclusion of vertebrate scavengers indeed resulted in higher concentrations of Cu and Zn below carcasses (Fig. 3b, f). This is also in line with previous studies (e.g. Parmenter and MacMahon 2009; Quaggiotto et al. 2019; Barton et al. 2020). For example, Quaggiotto et al. (2019) and Barton et al. (2020) excluded vertebrate scavengers but allowed invertebrates, and found increased EC, pH, and total N and P. Increased EC indicates increasing elemental concentrations but does not give information for any element separately (e.g. Wilson 1938; Heiniger et al. 2003; Selway 2014). In this paper, we followed eleven elements underneath carcasses over time.

We did not observe any clear differences among the three treatments that allowed different subsets of vertebrate scavengers. Only the treatment with only invertebrate scavengers showed some different patterns in Cu and Zn concentrations over time (Table 3). The treatments allowing vertebrates but no wild boar did not differ from the treatment allowing all scavengers, in disagreement with our expectation that wild boar would have a stronger influence than other vertebrates. Yet our findings of unaltered nutrient pools below carcasses are in line with Bump et al. (2009), who allowed all vertebrate scavengers and found no or only minor changes in local biochemical dynamics. Melis et al. (2007) also allowed all scavengers and concluded that large proportions of carcass-derived nutrients are recycled via these scavengers rather than locally leaking into the soil. Macdonald et al. (2014), in contrast, found increased EC beneath carcasses while allowing all scavengers, but did not elaborate on the degree to which vertebrates contributed to the decomposition process, hence it is not clear to which extent vertebrates consumed the carcasses. In general, elemental changes were not likely to be due to changes in edaphic properties like pH in our study (Appendix 4), implying that the changes that we found could be attributed to the carcasses.

Overall, fluxes of trace elements below carcasses seemed to be more influenced by invertebrate scavengers than by vertebrates since we only found elevated concentrations of trace elements when only invertebrate scavengers and microbial decomposers had access to the carcasses. Vertebrate scavengers seem to limit leakage of elements from carcasses into the soil. Particularly, we found that Cu levels increased in the soil and in the below-ground vegetation parts when only invertebrate scavengers were present (Figs. 3b, 5b). Cu is an essential trace element (Robinson et al. 2009; Kaspari 2021). It is a component in many enzymes in animals (e.g. NRC 2001) and plays a key role in photosynthesis in plants (e.g. Yruela 2009). Our results suggest that plants can immediately take up increased levels of Cu when it is supplied to the soil. The peak in Cu levels in the shoots (Fig. 7b) suggests that those plants can invest it in the above-ground vegetation parts as well. This seems to also apply for Zn (Figs. 3f, 5f, 7f), another essential element in both plants and animals (e.g. Broadley et al. 2007; Papachristodoulou et al. 2015).

Fluxes of macro elements were not significantly different between exclusion treatments. Yet the highest values in the soil for some of these elements, e.g. K, P and S (Fig. 4b, d, e), were found in the treatment excluding all vertebrates. This implies that these elements might leak into the soil during decomposition processes dominated by invertebrates but that our sample size might be too low to statistically confirm. This potential leakage was also found in controlled experiments (Wenting et al. 2023a).

We did not find any significant pattern for macro elements and most of the trace elements (except Cu and Zn) in any of the sample types. Apart from no elements leaking from carcasses, which is highly unlikely (Wenting et al. 2023a), a likely explanation for our results could be that essential elements are rapidly taken up by plants (Hobbie 1992). This could have resulted in an increase in biomass, rather than an increase of elemental concentrations in plants (e.g. Ingestad and Agren 1991; Pilon-Smits et al. 2009). This explanation seems to be in line with previous studies that described that elemental leakage due to carcass decomposition can enhance plant growth (e.g. Towne 2000; Wenting et al. 2023a). We were, however, unable to measure this alleged rapid uptake by plants and investment in biomass since we decided not to sample plant biomass in our experiment for several reasons. For instance, we sampled the carcasses repeatedly and chose not to disrupt the experiment drastically by clipping vegetation. In addition, our experimental setup did not allow us to prevent herbivory. Herbivory—both by vertebrates (e.g.,Bigger & Marvier 1998; Ford & Grace 1998) and invertebrates (e.g.,Carson & Root 1999; Belovsky & Slade 2000; Throop 2005; La Pierre et al. 2015)—can reduce plant biomass enormously. We believe it is worthwhile to examine this concept of plant biomass increase due to carcass-driven elemental fluxes and enhanced herbivory in a more controlled experiment.

Some essential scarce trace elements, e.g., Co (NRC 2001), showed higher concentrations in the control soil samples than in fallow deer bodies (Table 2). We could, therefore, not measure local changes for these elements that could be attributed to carcass decomposition. However, it has been demonstrated in a controlled experiment that such trace elements can also leak into the soil due to carcass decomposition (Wenting et al. 2023a). In a more natural setting, like the experiment we report here, Co might be hardly detectable due to its scarcity (e.g., Pratt and Fonstad 2009).

Most ecotoxic elements—Al, As, Cd, and Pb—were measured in higher concentrations in the control soil samples than in fallow deer bodies (Table 2). Al is poorly absorbed in animal bodies (NRC 2001; Pérez-Granados and Vaquero 2002), which might be reflected by our finding. Contrary, As is well absorbed by animals and toxicity is likely to occur (NRC 2001; Ventura-Lima et al. 2011). Pb is the most common cause of toxicoses in animals (Neathery and Miller 1975). Our findings imply that As and Pb toxicity might not be of great importance in our study area. Cd, a heavy metal without any function but causing severe renal damage (e.g.,Patra et al. 2006; Yu et al. 2006; Swarup et al. 2007), was the only ecotoxic element that we measured in lower concentrations in the soil control samples than in fallow deer. Although we can only speculate about the consequences of these findings, this might imply a non-excessive ecotoxic load in our study area. Therefore, the decomposition of carcasses is unlikely to worsen the overall toxicity in the landscape.

Overall, our results raise several new questions worth exploring. Our experimental design did not allow us to evaluate the role of invertebrate scavenger communities in changes in elemental concentrations below carcasses. In addition, the treatments allowing vertebrate scavengers did not lead to increased elemental concentrations, implying that vertebrates absorbed the elements in their bodies. We encourage further testing to understand how these scavengers utilize their habitats and disperse the absorbed elements across their landscape.

In conclusion, we found that vertebrate scavengers limited the influence of elemental leakage from the carcasses into the soil, and hence did not influence local nutrient dynamics and local soil nutrient pools. When vertebrate scavengers were excluded, fluxes of several nutrients into the soil showed distinct peaks, which was also reflected in the vegetation. Especially, essential trace elements (Cu and Zn) seemed to be influenced by carcass decomposition. However, we did not find any significant differences between treatments with partial exclusion of vertebrate scavengers. Although wild boar can significantly speed up the carcass decomposition process compared to other vertebrates (e.g., Wenting et al. 2022), this species does not seem to differently influence elemental leakage during the process. Our results suggest that carcass-derived nutrients are dispersed over larger areas rather than locally leak into the soil when vertebrate scavengers dominate the decomposition process. Hence, vertebrate scavengers spatially homogenize soil chemical elements in the landscape, in contrast to carcasses that are not consumed by vertebrate scavengers that result in islands of soil fertility.

## Supplementary Information

Below is the link to the electronic supplementary material.

Appendix 1 Photos of the experimental design: the transportable scaffolding used for lifting the carcasses (DOCX 400 KB)

Appendix 2 Test statistics and figures of the control samples (DOCX 1656 KB)

Appendix 3 Test statistics of the comparison of the initial elemental concentrations (DOCX 19 KB)

Appendix 4 Test statistics of the samples on the carcass sites (DOCX 152 KB)

## Data Availability

The complete data set used in this study is available through Figshare: https://doi.org/10.6084/m9.figshare.24865638
