# Supplementary material for: The influence of vertebrate scavengers on leakage of nutrients from carcasses
Source: Oecologia. 2024 Aug 17;206(1-2):21–35. doi: 10.1007/s00442-024-05608-w (PMC11489260; doi:10.1007/s00442-024-05608-w)
Supplement: Supplementary file 1 — Appendix 1 Photos of the experimental design: the transportable scaffolding used for lifting the carcasses (DOCX 400 KB) [file 442_2024_5608_MOESM1_ESM.docx]

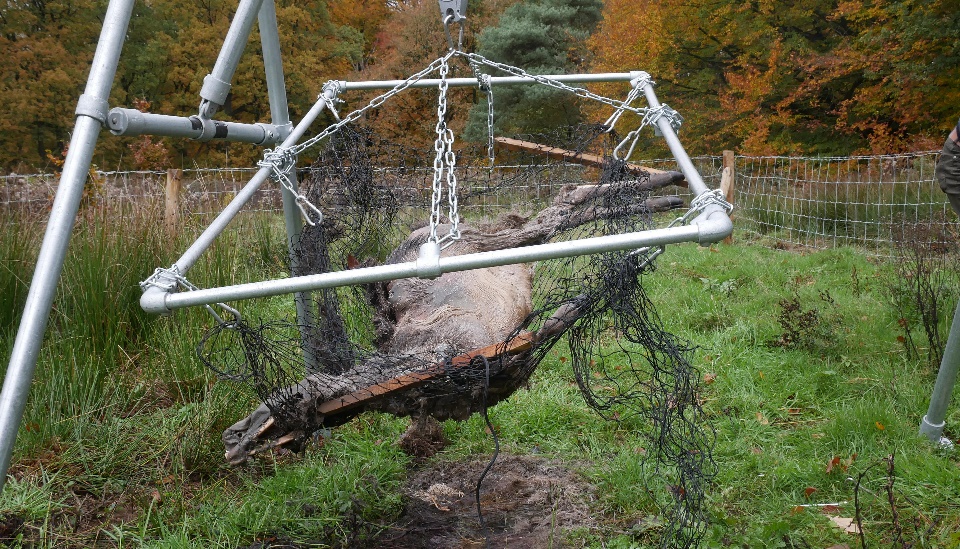


Figure S1.1 A carcass (treatment 2) in the transportable scaffolding. This was used to lift the carcasses (to access the soil) and to put them back in exactly the same position as before.
