# Supplementary material for: The influence of vertebrate scavengers on leakage of nutrients from carcasses
Source: Oecologia. 2024 Aug 17;206(1-2):21–35. doi: 10.1007/s00442-024-05608-w (PMC11489260; doi:10.1007/s00442-024-05608-w)
Supplement: Supplementary file 2 — Appendix 2 Test statistics and figures of the control samples (DOCX 1656 KB) [file 442_2024_5608_MOESM2_ESM.docx]

Table S2.1 Test statistics belonging to the LMMs of the control soil samples.

| Element | Sum of Squares | Numerator Degrees of Freedom | Denominator Degrees of Freedom | F value | Adjusted p value |
| --- | --- | --- | --- | --- | --- |
| Al | 3485740 | 3 | 243.104 | 0.327 | 0.979 |
| As | 2.056231 | 3 | 244.833 | 0.383 | 0.979 |
| B | 6.945086 | 3 | 244.383 | 0.518 | 0.979 |
| Ca | 903828.9 | 3 | 244.422 | 0.659 | 0.979 |
| Cd | 0.036743 | 3 | 242.738 | 0.432 | 0.979 |
| Co | 11.4317 | 3 | 243.974 | 0.935 | 0.979 |
| Cr | 16.53621 | 3 | 239.057 | 0.315 | 0.979 |
| Cu | 110.9044 | 3 | 236.481 | 1.005 | 0.979 |
| Fe | 5181358 | 3 | 241.988 | 0.515 | 0.979 |
| K | 1423367 | 3 | 244.174 | 0.064 | 0.979 |
| Mg | 108343.8 | 3 | 243.206 | 0.312 | 0.979 |
| Mn | 170708.5 | 3 | 245.703 | 0.647 | 0.979 |
| Mo | 0.816257 | 3 | 243.207 | 0.112 | 0.979 |
| Na | 6744.663 | 3 | 101.940 | 0.493 | 0.979 |
| Ni | 2.661034 | 3 | 244.002 | 0.356 | 0.979 |
| P | 85617.54 | 3 | 245.658 | 0.082 | 0.979 |
| Pb | 605.1686 | 3 | 244.172 | 0.731 | 0.979 |
| S | 88618.68 | 3 | 243.915 | 0.182 | 0.979 |
| Se | 2.538428 | 3 | 197.200 | 0.696 | 0.979 |
| Si | 4189220 | 3 | 244.099 | 0.999 | 0.979 |
| Sr | 37.58342 | 3 | 245.298 | 2.016 | 0.979 |
| Zn | 4355.319 | 3 | 239.713 | 0.313 | 0.979 |

Table S2.2 Test statistics belonging to the LMMs of the control root samples.

| Element | Sum of Squares | Numerator Degrees of Freedom | Denominator Degrees of Freedom | F value | Adjusted p value |
| --- | --- | --- | --- | --- | --- |
| Al | 14163854 | 3 | 245.709 | 1.012 | 0.547 |
| As | 3.136911 | 3 | 240.611 | 0.654 | 0.673 |
| B | 30.7333 | 3 | 240.780 | 0.990 | 0.547 |
| Ca | 10906357 | 3 | 244.946 | 1.954 | 0.547 |
| Cd | 2.540212 | 3 | 244.702 | 1.529 | 0.547 |
| Co | 5.757023 | 3 | 240.583 | 1.790 | 0.547 |
| Cr | 54.61888 | 3 | 243.395 | 1.090 | 0.547 |
| Cu | 202.0703 | 3 | 243.655 | 1.837 | 0.547 |
| Fe | 14739290 | 3 | 245.195 | 1.005 | 0.547 |
| K | 97925513 | 3 | 244.292 | 1.022 | 0.547 |
| Mg | 1100887 | 3 | 244.918 | 1.282 | 0.547 |
| Mn | 546403.1 | 3 | 244.445 | 0.575 | 0.695 |
| Mo | 11.4177 | 3 | 235.873 | 0.739 | 0.648 |
| Na | 312368 | 3 | 224.702 | 1.207 | 0.547 |
| Ni | 23.42884 | 3 | 243.213 | 0.874 | 0.589 |
| P | 3415026 | 3 | 244.569 | 1.254 | 0.547 |
| Pb | 317.4546 | 3 | 244.424 | 0.400 | 0.753 |
| S | 2523767 | 3 | 244.142 | 1.458 | 0.547 |
| Se | 6.540606 | 3 | 174.751 | 1.027 | 0.547 |
| Si | 14336349 | 3 | 243.213 | 0.435 | 0.753 |
| Sr | 99.80077 | 3 | 245.419 | 1.448 | 0.547 |
| Zn | 51654.77 | 3 | 245.241 | 1.323 | 0.547 |

Table S2.3 Test statistics belonging to the LMMs of the control shoot samples.

| Element | Sum of Squares | Numerator Degrees of Freedom | Denominator Degrees of Freedom | F value | Adjusted p value |
| --- | --- | --- | --- | --- | --- |
| Al | 2031125 | 3 | 236.000 | 0.280 | 0.965 |
| As | 0.472925 | 3 | 233.000 | 0.173 | 0.965 |
| B | 51.45427 | 3 | 217.064 | 0.799 | 0.965 |
| Ca | 7126969 | 3 | 231.014 | 0.510 | 0.965 |
| Cd | 1.243894 | 3 | 229.507 | 1.549 | 0.965 |
| Co | 1.207697 | 3 | 226.792 | 0.709 | 0.965 |
| Cr | 20.60693 | 3 | 229.508 | 0.499 | 0.965 |
| Cu | 79.73795 | 3 | 231.240 | 0.344 | 0.965 |
| Fe | 2328665 | 3 | 231.937 | 0.291 | 0.965 |
| K | 6.78E+08 | 3 | 231.400 | 1.585 | 0.965 |
| Mg | 464057.5 | 3 | 231.237 | 0.261 | 0.965 |
| Mn | 922685 | 3 | 230.778 | 0.807 | 0.965 |
| Mo | 50.2785 | 3 | 229.958 | 1.207 | 0.965 |
| Na | 67138.73 | 3 | 151.030 | 0.975 | 0.965 |
| Ni | 1.264184 | 3 | 233.263 | 0.130 | 0.965 |
| P | 4879887 | 3 | 231.264 | 0.861 | 0.965 |
| Pb | 199.5373 | 3 | 228.627 | 0.365 | 0.965 |
| S | 402333.7 | 3 | 232.011 | 0.092 | 0.965 |
| Se | 2.17702 | 3 | 182.483 | 0.534 | 0.965 |
| Si | 3.78E+08 | 3 | 234.000 | 0.654 | 0.965 |
| Sr | 97.55687 | 3 | 229.786 | 0.728 | 0.965 |
| Zn | 18337.59 | 3 | 230.189 | 0.598 | 0.965 |


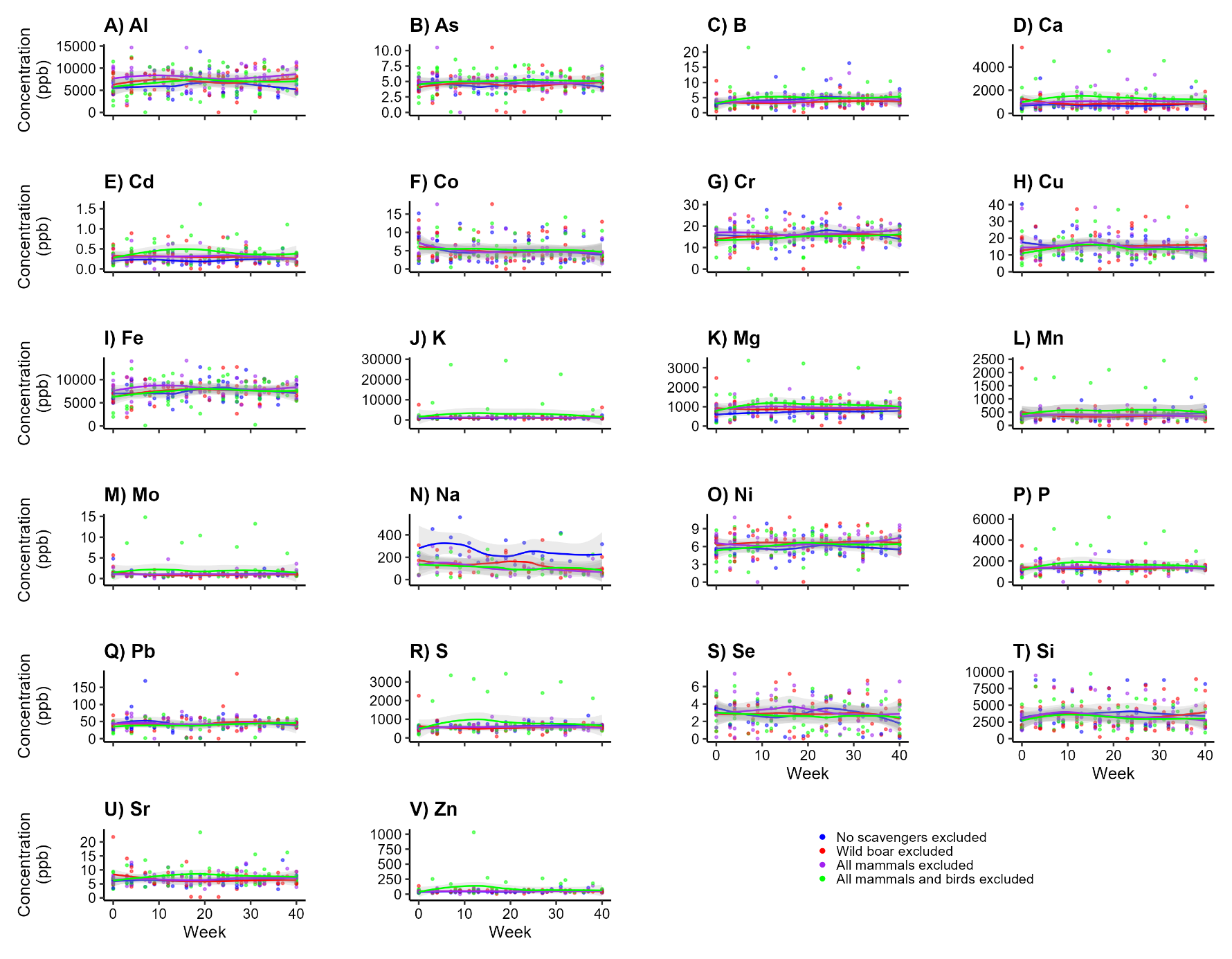


Fig. S2.1 Elemental concentrations of the control soil samples.


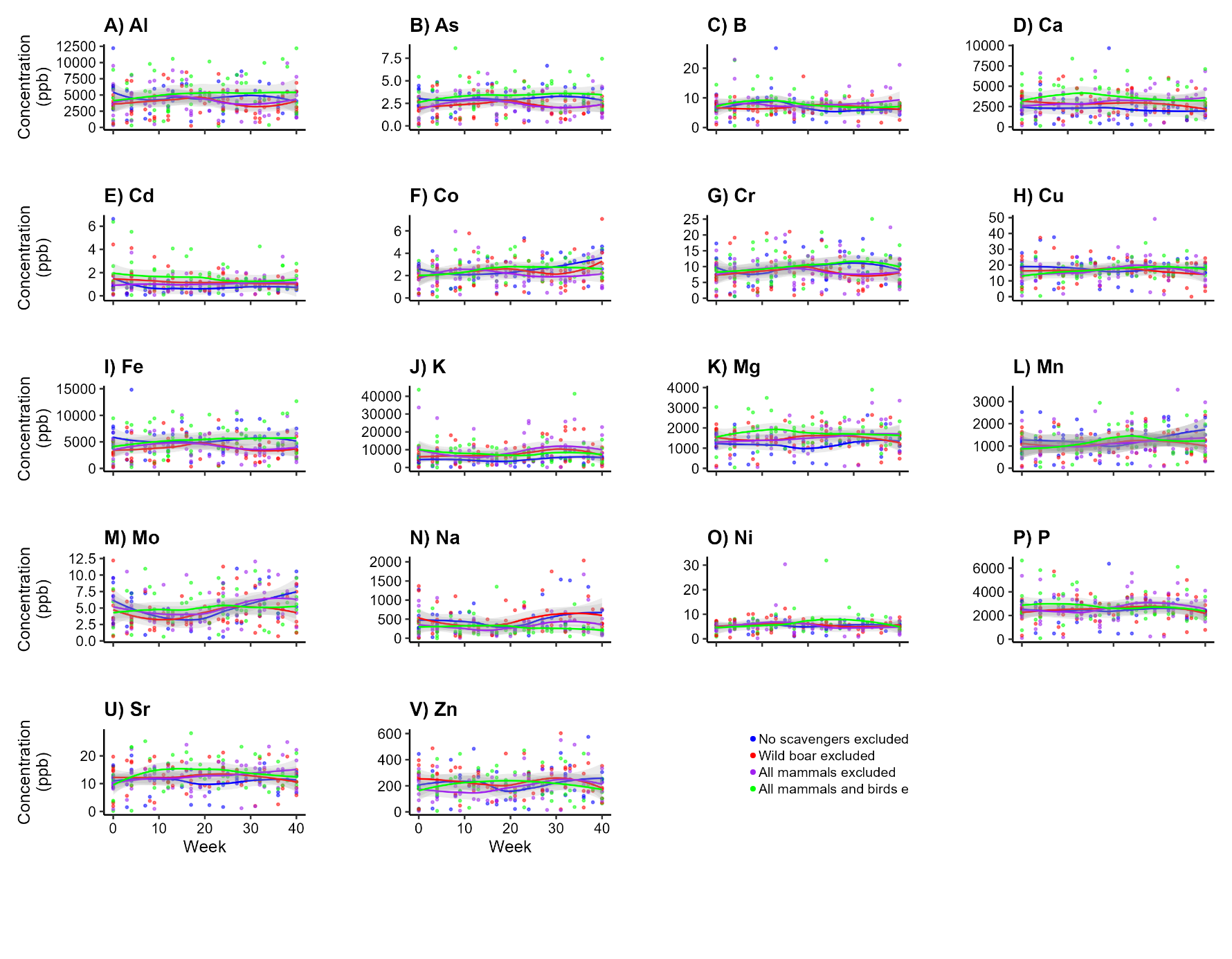


Fig. S2.2 Elemental concentrations of the control root samples.


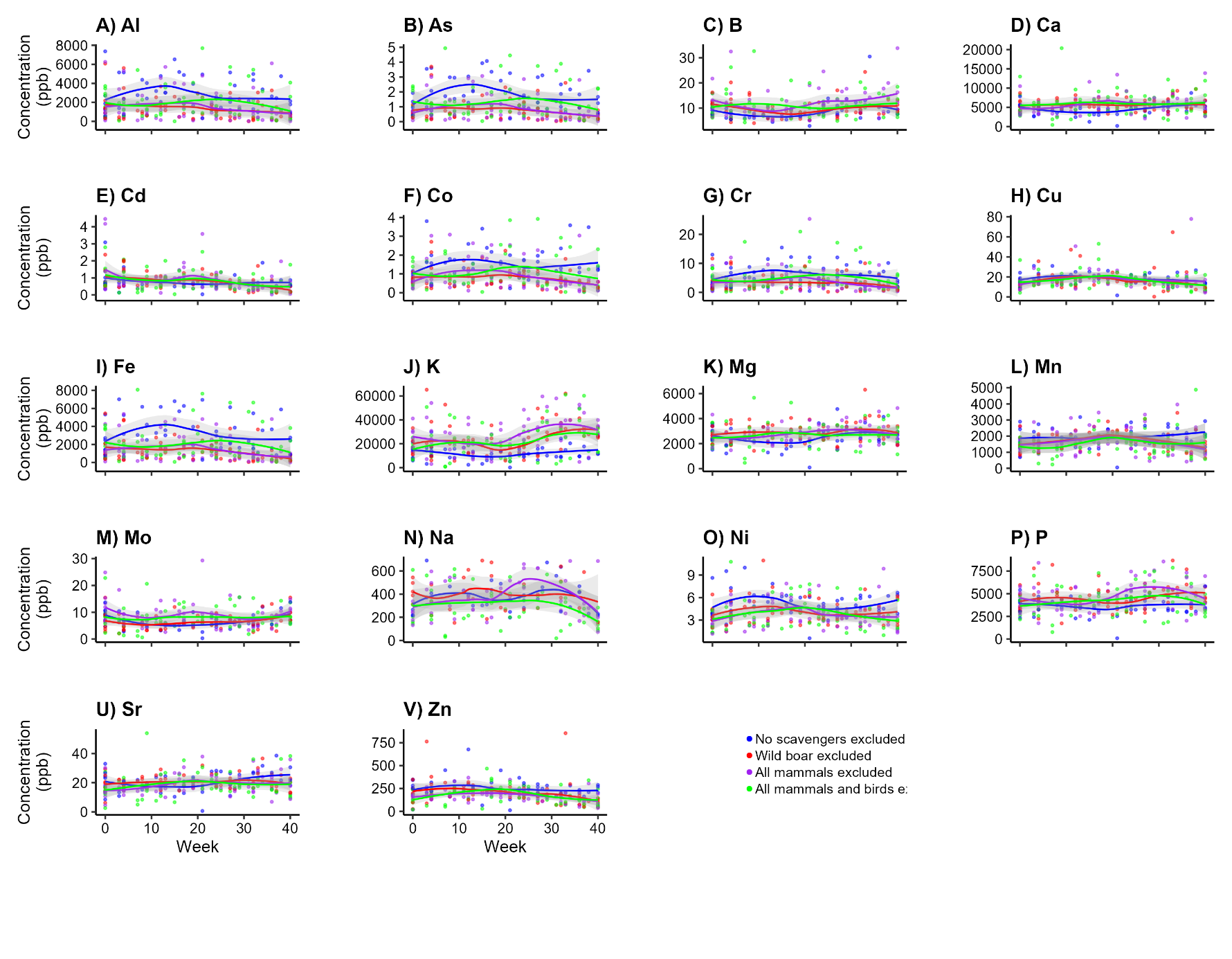


Fig. S2.3 Elemental concentrations of the control shoot samples.
