# Supplementary material for: The influence of vertebrate scavengers on leakage of nutrients from carcasses
Source: Oecologia. 2024 Aug 17;206(1-2):21–35. doi: 10.1007/s00442-024-05608-w (PMC11489260; doi:10.1007/s00442-024-05608-w)
Supplement: Supplementary file 3 — Appendix 3 Test statistics of the comparison of the initial elemental concentrations (DOCX 19 KB) [file 442_2024_5608_MOESM3_ESM.docx]

Table S3.1 Test statistics belonging to the Mann-Whitney U tests of the initial concentrations in the soil samples.

| Element | W | Adjusted p value |
| --- | --- | --- |
| B | 468 | 0.691 |
| Ca | 462 | 0.691 |
| Cu | 462 | 0.691 |
| K | 432 | 0.877 |
| Mg | 458 | 0.691 |
| Mn | 431 | 0.877 |
| Mo | 511 | 0.691 |
| Ni | 459 | 0.691 |
| P | 461 | 0.691 |
| S | 459 | 0.691 |
| Zn | 461 | 0.691 |

Table S3.2 Test statistics belonging to the Mann-Whitney U tests of the initial concentrations in the root samples.

| Element | W | Adjusted p value |
| --- | --- | --- |
| B | 431 | 0.943 |
| Ca | 408 | 0.943 |
| Cu | 380 | 0.943 |
| K | 464 | 0.943 |
| Mg | 413 | 0.943 |
| Mn | 401 | 0.943 |
| Mo | 355 | 0.943 |
| Ni | 316 | 0.943 |
| P | 447 | 0.943 |
| S | 435 | 0.943 |
| Zn | 410 | 0.943 |

Table S3.3 Test statistics belonging to the Mann-Whitney U tests of the initial concentrations in the shoot samples.

| Element | W | Adjusted p value |
| --- | --- | --- |
| B | 302 | 0.733 |
| Ca | 360 | 0.945 |
| Cu | 511 | 0.118 |
| K | 404.5 | 0.777 |
| Mg | 369 | 0.945 |
| Mn | 414 | 0.733 |
| Mo | 382 | 0.942 |
| Ni | 398 | 0.785 |
| P | 414 | 0.733 |
| S | 435 | 0.733 |
| Zn | 438 | 0.733 |
