# Supplementary material for: The influence of vertebrate scavengers on leakage of nutrients from carcasses
Source: Oecologia. 2024 Aug 17;206(1-2):21–35. doi: 10.1007/s00442-024-05608-w (PMC11489260; doi:10.1007/s00442-024-05608-w)
Supplement: Supplementary file 4 — Appendix 4 Test statistics of the samples on the carcass sites (DOCX 152 KB) [file 442_2024_5608_MOESM4_ESM.docx]

Table S4.1 Test statistics belonging to the interaction terms of treatment and sampling week of the the LMMs of the soil samples beneath the carcasses.

| Element | Sum of Squares | Numerator Degrees of Freedom | Denominator Degrees of Freedom | F value | Adjusted p value |
| --- | --- | --- | --- | --- | --- |
| B | 0.577507 | 3 | 258.210 | 0.587 | 0.993 |
| Ca | 0.86183 | 3 | 259.473 | 0.420 | 0.993 |
| Cu | 21.81054 | 3 | 252.529 | 5.917 | 0.004 |
| K | 5.013046 | 3 | 260.122 | 0.040 | 0.993 |
| Mg | 0.042968 | 3 | 259.226 | 0.029 | 0.993 |
| Mn | 0.110249 | 3 | 259.673 | 0.032 | 0.993 |
| Mo | 0.239433 | 3 | 259.644 | 0.051 | 0.993 |
| Ni | 0.638478 | 3 | 258.516 | 1.357 | 0.940 |
| P | 1.230962 | 3 | 259.139 | 0.550 | 0.993 |
| S | 2.126002 | 3 | 259.683 | 0.537 | 0.993 |
| Zn | 482.2165 | 3 | 259.013 | 7.795 | <0.001 |

Table S4.1.1 Test statistics belonging to the Tukey post hoc tests of the statistically significant elements in Table S4.1.

|  |  | Estimate | SE | df | t.ratio | p value |
| --- | --- | --- | --- | --- | --- | --- |
| Element: Cu |  |  |  |  |  |  |
| Treatment 1 | Treatment 2 | -0.211 | 0.207 | 256 | -1.020 | 0.738 |
| Treatment 1 | Treatment 3 | 0.054 | 0.204 | 257 | 0.264 | 0.994 |
| Treatment 1 | Treatment 4 | -1.251 | 0.203 | 256 | -6.171 | <0.001 |
| Treatment 2 | Treatment 3 | 0.265 | 0.188 | 253 | 1.409 | 0.495 |
| Treatment 2 | Treatment 4 | -1.040 | 0.188 | 252 | -5.532 | <0.001 |
| Treatment 3 | Treatment 4 | -1.305 | 0.184 | 252 | -7.104 | <0.001 |
| Element: Zn |  |  |  |  |  |  |
| Treatment 1 | Treatment 2 | -0.270 | 0.833 | 264 | -0.324 | 0.988 |
| Treatment 1 | Treatment 3 | -0.381 | 0.829 | 265 | -0.459 | 0.968 |
| Treatment 1 | Treatment 4 | -6.025 | 0.829 | 264 | -7.269 | <0.001 |
| Treatment 2 | Treatment 3 | -0.111 | 0.753 | 259 | -0.147 | 0.999 |
| Treatment 2 | Treatment 4 | -5.755 | 0.757 | 258 | -7.599 | <0.001 |
| Treatment 3 | Treatment 4 | -5.644 | 0.747 | 258 | -7.555 | <0.001 |

Table S4.2 Test statistics belonging to the interaction terms of treatment and sampling week of the LMMs of the root samples on the edge of the carcasses.

| Element | Sum of Squares | Numerator Degrees of Freedom | Denominator Degrees of Freedom | F value | Adjusted p value |
| --- | --- | --- | --- | --- | --- |
| B | 54.14095 | 3 | 255.390 | 0.303 | 0.906 |
| Ca | 41.50442 | 3 | 255.192 | 1.191 | 0.575 |
| Cu | 248.2412 | 3 | 239.049 | 7.234 | <0.001 |
| K | 2076.519 | 3 | 255.020 | 1.609 | 0.575 |
| Mg | 36.21557 | 3 | 255.029 | 0.948 | 0.657 |
| Mn | 3112.679 | 3 | 256.796 | 0.007 | 0.999 |
| Mo | 10.00443 | 3 | 251.365 | 0.690 | 0.769 |
| Ni | 14.53721 | 3 | 252.024 | 1.310 | 0.575 |
| P | 70.89681 | 3 | 255.040 | 2.015 | 0.575 |
| S | 144.1816 | 3 | 256.041 | 1.197 | 0.575 |
| Zn | 324.5851 | 3 | 253.847 | 0.347 | 0.906 |

Table S4.2.1 Test statistics belonging to the Tukey post hoc tests of the statistically significant elements in Table S4.2.

|  |  | Estimate | SE | df | t.ratio | p value |
| --- | --- | --- | --- | --- | --- | --- |
| Element: Cu |  |  |  |  |  |  |
| Treatment 1 | Treatment 2 | 0.369 | 0.627 | 240 | 0.588 | 0.936 |
| Treatment 1 | Treatment 3 | 0.330 | 0.630 | 241 | 0.523 | 0.953 |
| Treatment 1 | Treatment 4 | -2.751 | 0.652 | 240 | -4.220 | <0.001 |
| Treatment 2 | Treatment 3 | -0.039 | 0.573 | 239 | -0.068 | 0.999 |
| Treatment 2 | Treatment 4 | -3.119 | 0.598 | 239 | -5.212 | <0.001 |
| Treatment 3 | Treatment 4 | -3.080 | 0.594 | 239 | -5.185 | <0.001 |

Table S4.3 Test statistics belonging to the interaction terms of treatment and sampling week of the the LMMs of the shoot samples on the edge of the carcasses.

| Element | Sum of Squares | Numerator Degrees of Freedom | Denominator Degrees of Freedom | F value | Adjusted p value |
| --- | --- | --- | --- | --- | --- |
| B | 1.347867 | 3 | 242.822 | 1.631 | 0.287 |
| Ca | 1.000824 | 3 | 245.073 | 1.717 | 0.287 |
| Cu | 14.08744 | 3 | 241.221 | 0.709 | 0.602 |
| K | 27.80677 | 3 | 245.036 | 2.418 | 0.184 |
| Mg | 1.406854 | 3 | 246.009 | 3.570 | 0.054 |
| Mn | 0.566442 | 3 | 245.842 | 1.051 | 0.453 |
| Mo | 1.77177 | 3 | 244.941 | 3.896 | 0.053 |
| Ni | 1.280269 | 3 | 243.979 | 1.207 | 0.423 |
| P | 5.027666 | 3 | 244.859 | 4.062 | 0.053 |
| S | 4.784123 | 3 | 245.271 | 1.714 | 0.287 |
| Zn | 6.093634 | 3 | 243.778 | 0.050 | 0.985 |


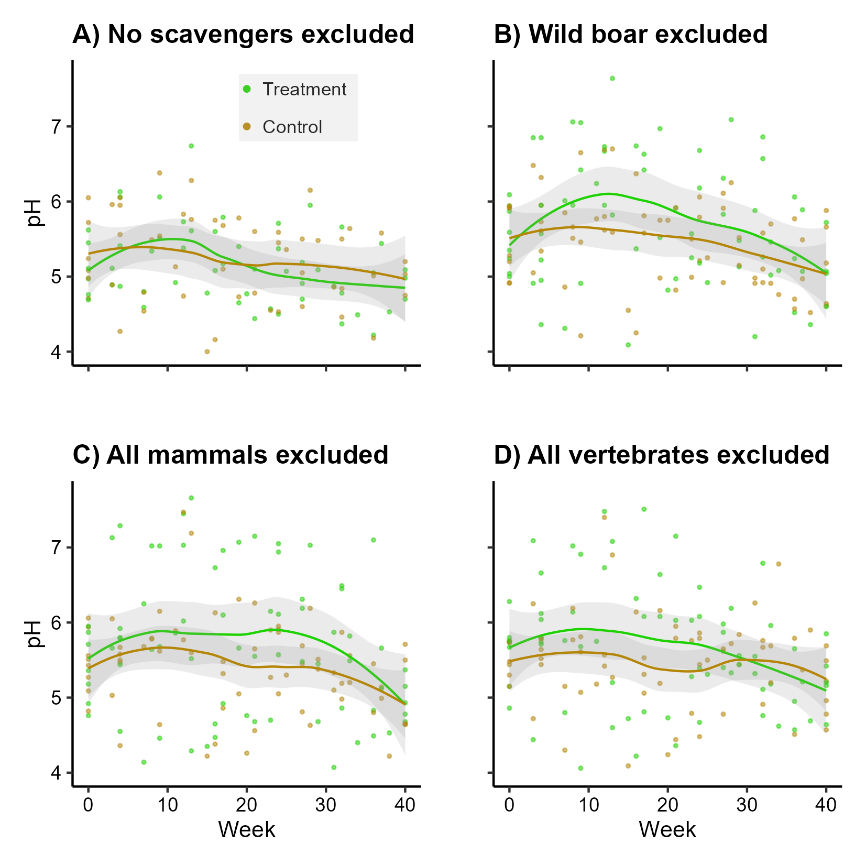


Fig. S4.1 Soil pH over time per scavenger exclusion treatment.

Table S4.4 Test statistics belonging to the interaction terms of treatment and sampling week of the the LMMs of the soil pH.

| Treatment | Sum of Squares | Numerator Degrees of Freedom | Denominator Degrees of Freedom | F value | p value |
| --- | --- | --- | --- | --- | --- |
| Treatment 1 | 0.00229 | 1 | 97.058 | 0.0198 | 0.888 |
| Treatment 2 | 0.00024 | 1 | 125.04 | 0.0010 | 0.975 |
| Treatment 3 | 0.00489 | 1 | 130.92 | 0.0134 | 0.908 |
| Treatment 4 | 0.93792 | 1 | 128.84 | 2.9003 | 0.091 |
